# PP96 Evaluating The Quality-Of-Life Impact Of Erythropoiesis-Stimulating Agents For Lower Risk (Low To Intermediate-1) Myelodysplastic Syndrome Patients

**DOI:** 10.1017/S0266462324002605

**Published:** 2025-01-07

**Authors:** Alastair Bennett, Andrea Manca

## Abstract

**Introduction:**

Patients diagnosed with low- to intermediate-1-risk myelodysplastic syndrome (LR-MDS) encounter symptom burden that diminishes their health-related quality of life (HRQoL). Erythropoiesis-stimulating agents (ESAs) remain an option to alleviate anemia-related symptoms. However, existing HRQoL studies show limited evidential support. This study assesses the impact of ESAs on LR-MDS patients’ EuroQol 5-dimension questionnaire (EQ-5D) scores compared to not using ESA as initial therapy.

**Methods:**

The European MDS Registry (EUMDS) collects information including ESA usage, covariates, and EQ-5D scores at six-month intervals. Estimating average treatment effect (ATE) from observational data requires adjusting for several sources of bias. Our study controls for baseline and time-varying confounding by using inverse probability of treatment weights. Employing a methodology based on marginal structural models, we are able to estimate robust ATEs. A two-part mixed-effects beta model was used to calculate ATE during a four-year follow-up period. We compare ESA therapy every six months versus clinical management not involving the use of ESAs.

**Results:**

Our results show an overall positive ESA effect on EQ-5D over the four-year follow-up period. The majority of time points have a positive ESA effect after adjustment, though a few time points show no effect. The estimated ATE at four years is small: 0.046 (−0.031, 0.114).

**Conclusions:**

We found that use of ESAs over a four-year follow-up period produces mostly positive treatment effect estimates after adjusting for time-varying variables and confounders. Our robust results can be used to inform more reliable treatment decision-making.

